# High rates of early HBeAg seroconversion and relapse in Indian patients of chronic hepatitis B treated with Lamivudine: results of an open labeled trial

**DOI:** 10.1186/1471-230X-5-29

**Published:** 2005-09-15

**Authors:** George Alexander, Chalamalasetty S Baba, Kamal Chetri, TS Negi, Gourdas Choudhuri

**Affiliations:** 1 Department of Gastroenterology, Sanjay Gandhi Postgraduate Institute of Medical Sciences, Lucknow, India

## Abstract

**Background:**

The use of Lamivudine in chronic hepatitis B (CHB) is well known, however the reported rate of HBeAg sero-conversion and its durability post-treatment have varied considerably. We undertook the present study to study the effect of Lamivudine on HBeAg loss and seroconversion rates in Indian patients of CHB in relation to frequency, predictors and durability.

**Methods:**

We treated 60 patients of e antigen positive CHB (with active viral replication and ongoing necro-inflammatory activity) with Lamivudine. They were followed up by monthly aminotransferases, and 3 monthly HBeAg and anti-HBe. Those who attained HBeAg sero-conversion were advised to discontinue Lamivudine after 6 months and followed up every 3 months thereafter, to see for relapse. Treatment was given for maximum of 3 years if not sero-converted.

**Results:**

The annual incremental loss of HBeAg in patients receiving Lamivudine was 25 (41.6%) at end of 1^st ^year, 33 (55%) at 2^nd ^year and 35 (58.3%) at 3^rd ^year. The corresponding rates for full sero-conversion were 17/60 (28.6%), 22/60 (36.6%) and 24/60 (40%) in the 3 years. HBeAg loss correlated with increased pre-therapy ALT levels (p = 0.002) and decreased pretreatment HBV-DNA levels (p = 0.004). The presence of cirrhosis had no influence on the rate of HBeAg loss. Relapse occurred in 35% (7/20) post-treatment at median time of 6 months.

**Conclusion:**

Indian patients showed a higher rate of HBeAg sero-conversion in the first year of Lamivudine treatment. This correlated with baseline ALT and inversely with HBV-DNA levels. Relapse rate after treatment was high and occurred soon after stopping treatment.

## Background

Hepatitis B virus infects more than 300 million people worldwide, contributing to debilitating illness and death, and more than 75% of those affected are of Asian origin [1]. Chronically infected persons with viral replication are at highest risk for progressive liver disease and it is estimated that up to 25% of persons with chronic hepatitis B virus infection will die prematurely of cirrhosis or hepatocellular carcinoma. Cirrhosis and hepatocellular carcinoma account for more than 50% of deaths in Asian men with chronic Hepatitis B infection [2].

The risk of chronicity after acute HBV infection is low in immuno-competent adults and is reported to be less than 5%[3]. However, the natural history of HBV infection differs between Asian and Western patients. Asians usually become infected perinatally, rarely have an acute hepatitis-like clinical syndrome but almost invariably remain chronically infected and run substantial risk for developing cirrhosis and hepatocellular carcinoma. Western patients who are usually infected in adult life by percutaneous or sexual exposure, typically have an acute hepatitis-like clinical illness, clear the virus and rarely become chronically infected [4,5]. Chronically infected persons with evidence of active viral replication are at highest risk for progressive liver disease. Cirrhosis develops in 15 to 20 % of them within 5 years, even if histologic liver damage is initially mild [6]. HBeAg seroconversion, which can occur spontaneously or post-treatment, is associated with a substantial reduction in the risk of liver failure [7]. Spontaneous sero-conversion occurs only in 7 to 20 % per year [8].

Interferon -alfa was the first drug specifically licensed to treat chronic Hepatitis B. The efficacy of Interferon-alfa was found to be variable, but a meta-analysis showed that 33% receiving it lost HBeAg compared with 12% of untreated controls [9]. Interferon-alfa was least effective in Asian patients [10]. It has to be given by injection and has potential dose-limiting side effects. Patients with liver cirrhosis often deteriorated on initiating treatment [7]. Enthusiasm with Interferon for treatment for chronic hepatitis B therefore waned with the wide availability and use of Lamivudine.

Lamivudine, an oral nucleoside analogue, inhibits viral DNA replication. In doses of 100 mg/day in adults (1.5 mg/Kg/day in children), the median suppression of serum HBV-DNA is greater than 98% in most patients during treatment [11]. It produces rapid decrease in serum HBV- DNA and aminotransferase levels, improves liver histology and enhances the rate of HBeAg loss compared with placebo treatment [12,13]. The virologic end points of treatment have been the sustained disappearance of serum HBV DNA by a conventional hybridization assay and either the disappearance of hepatitis B e antigen from serum [HBeAg loss] or the loss of HBeAg accompanied by the detection of anti-HBe [HBeAg sero-conversion] [11,12]. The proportion of patients achieving HBeAg sero-conversion after 12 months of Lamivudine treatment [100 mg/day] has ranged between 16% and 18% in Western and Asian studies [11-13]. Several workers have shown pre-treatment high serum ALT and low serum HBV-DNA levels to be independently associated with increased rate of HBeAg loss and sero-conversion when treated with Lamivudine [13,14].

The safety of Lamivudine has led to the suggestion that continuous therapy may be beneficial, particularly in patients who do not lose HBeAg. Extended treatment with Lamivudine beyond 1 year has shown good results in various studies with results showing an incremental response in HBeAg sero-conversion rate [15,16]. The study by Leung NW et al showed sero-conversion rates of 22% after 1 year, increasing to 29% after 2 years and 40% after 3 years [16]. A major limitation of chronic therapy however is the development of viral resistance, marked virologically by rise in HBV DNA levels despite continuation of therapy and clinically by increases in serum transaminases [11,12,15,17]. Lamivudine induced HBeAg seroconversion was reported to be durable by several Western studies, 80 to 90%[12] and 73%[18]. However, studies in Asian countries show that relapse rates are much higher post treatment, 37.5% at 1 year [19] and 45.8%[20].

It has been observed that Hepatitis B virus may behave differently in different geographic regions [19,21]. This may be due to some host factors or to the viral genotypic differences [21]. Therefore it is important for each country to determine to determine its own rate and pattern of seroconversion following treatment of chronic hepatitis B. The aims of the present study was to study the effect of Lamivudine on chronic hepatitis B in Indian patients with regard to 1) rate of seroconversion and HBeAg loss 2) predictors of seroconversion 3) the durability of seroconversion post treatment.

## Methods

### Patients

Sixty patients [50 men and 10 women, median age 40 years, range 4–80 years, cirrhosis 23] with HBeAg positive chronic HBV infection who were started on Lamivudine 100 mg per day (1.5 mg/Kg/day in children) during the time period from August 1998 to June 2001 were followed up. All patients were positive for HBsAg for more than six months, had active replicative status [HBeAg positive, AntiHBe negative and HBV-DNA positive by PCR technique] with alanine aminotransferase levels that were less than 10 times the upper limit of normal for at least the previous 3 months. All had evidence of ongoing necro-inflammatory activity either on liver biopsy (knodell ishak score>4) or suggested by raised alanine aminotransferase levels. Cirrhosis was diagnosed on basis of clinical [evidence of portal hypertension, liver decompensation], biochemical, endoscopic [varices] and imaging [ultrasound] evidence. Patients were excluded if they had any of the following: previous antiviral treatment for hepatitis B; immunomodulatory drugs or corticosteroids within 6 months before Lamivudine treatment; co infection with hepatitis C virus or the human immunodeficiency virus; or the presence of other types of liver disease.

The study was approved by the local ethics committee and all patients provided informed consent before treatment.

### Methods

Lamivudine was given at a dose of 100 mg/day during the study period. Serum ALT was checked monthly and serum HBeAg, anti-HBe [measured by the commercially available ELISA kit; Organon Teknika] 3 monthly till seroconversion. HBV DNA testing by the In-house PCR technique was done to confirm viral suppression, initially at 3 months and then annually. It was also done at the time of seroconversion or biochemical breakthrough. HBV-DNA levels were done by Quantiplex branched DNA assay at baseline and at 3 months. HBeAg seroconversion was defined as disappearance of HBeAg and appearance of anti-HBe antibody, while HBeAg loss was defined as disappearance of HBeAg only. Viral breakthrough was identified on the basis of ALT rise (greater than 2 × upper limit of normal) with re-emergence of HBV-DNA positivity (by PCR) and detectable levels of HBV DNA. HBeAg seroconversion was confirmed by repeat testing after 3 months. Lamivudine was continued for 6 months after seroconversion was achieved or for maximum of 3 years. Thereafter post treatment monitoring in those who had seroconverted continued by monthly ALT and 3 monthly HBeAg, anti- HBe. This was done to look for relapse, which was defined as re-emergence of HBV DNA positivity by PCR technique and/or HBeAg positivity after Lamivudine was stopped post seroconversion.

### Statistics

The baseline factors evaluated were age, sex, BMI, weight, ALT, HBV DNA levels and presence of cirrhosis. Data were expressed as median (range) or mean+/- SD. For statistical significance, nominal variables were analyzed by Chi square test with Yates correction. For numerical variables Wilcoxon Rank Sum Test was used, as the data was not expected to have a Gaussian distribution. A p value of < 0.05 was taken as significant.

## Results

### Patient population

Sixty patients who received Lamivudine were followed up. All of them completed at least 1 year of treatment. In the second year 4 patients who had not seroconverted, did not come for follow up and similarly in the 3 rd year 6 patients dropped out. [See table 1 for baseline characteristics] The majority, 50 [83.3%], were men, and median age was 40 years [range 4–80 years]. The mode of transmission of HBV was unknown in most [61.7%] with blood transfusion history being present in 11.7% and history of hemodialysis in 13.3%. Elevated ALT was present in 50 [83.3%] of the 60 patients. The median ALT level was 72 U/L [range 27 to 394] and median HBV DNA level 920 [range: 0.8–4500] mEq/ml. The median × Upper limit of normal of ALT was 1.8. Cirrhosis was present in 23 [38.3%] of the patients.

### HBeAg response

**Figure 1:**
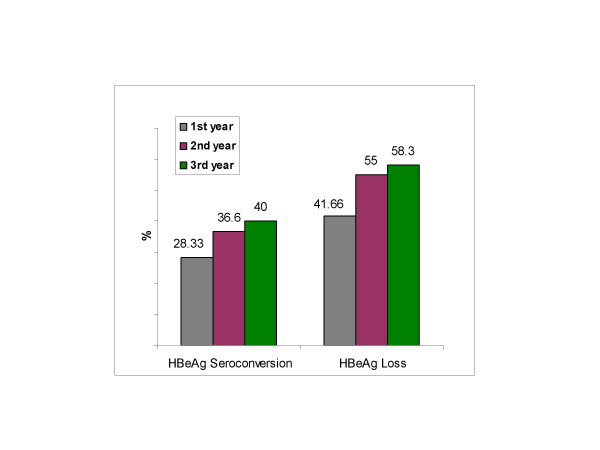
Cumulative HBeAg seroconversion and loss in 60 patients.

Sero-conversion of HBeAg to anti HBe occurred in 17 of 60 patients (28.33%) at the end of first year and incrementally rose to 36.6%(22/60) by second year and to 40%(24/60) by third year as shown in Figure 1. Of all sero-conversions maximum occurred in the first year (17/24) 71%, 21% in the 2nd year and only 8% in the 3rd year. The rate of HBeAg loss was 41.66%(25/60) in first year and incrementally rose to 58.3% (35/60) by third year of treatment. Similar to that seen for seroconversion, maximum loss of HBeAg occurred in the 1^st ^year (71.42%) as compared to 2^nd ^and 3^rd ^years of treatment (22.8% and 5.71 % respectively). Onset of seroconversion occurred at a mean of 10.17 months after starting treatment.

### Pretreatment factors influencing HBeAg loss

**Figure 2:**
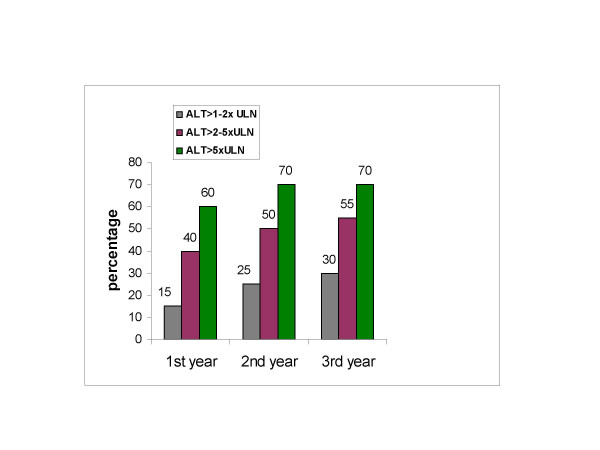
HBeAg Seroconversion Year wise According to Baseline ALT.

The association of age, sex, weight, BMI, baseline ALT, baseline HBV-DNA level and presence of cirrhosis with HBeAg loss was analyzed. [See table 2] Only baseline ALT and HBV DNA level were associated significantly with HBeAg clearance, with median ALT among those who lost their HBeAg being 94 U/L compared with median ALT in those who did not, 45 U/L (p = 0.002). Likewise the median HBV DNA level was 111.3 mEq/ml among those who lost their HBeAg as compared to that in those who did not, 958 mEq/ml (p = 0.004). Table 3 depicts the frequency of HBeAg loss and seroconversion according to baseline ALT level. HBeAg response rates increased with increase in level of pretreatment ALT. Among patients with pretreatment ALT levels greater than 1 to 2 times the ULN, HBeAg loss occurred in 50%, which increased to 70% among those with ALT levels greater than 2 to 5 times the ULN. The rate of HBeAg loss was highest among those with ALT levels greater than 5 times ULN, occurring in 80 %. Similar trends were observed with HBeAg seroconversion, though seroconversion occurred less frequently than HBeAg loss. Figure 2 depicts the HBeAg seroconversion rates year wise according to baseline ALT levels. After 1 year, 15% (3 of 20) of patients with baseline serum ALT >1–2 × ULN, 40% (8 of 20) with ALT>2 – 5 × ULN and 60% (6 of 10) had achieved seroconversion, increasing to 30% (6 of 20), 55% (11 of 20) and 70% (7 of 10) respectively after 3 years of treatment.

### ALT normalization and HBV-DNA levels

Alanine aminotransferase normalization occurred in 56% (28/50) of patients with elevated baseline ALT levels. This occurred more significantly in those who cleared their HBeAg (71.8%) than those who did not (27.7%), p = 0.02 [see table 2] Among the 24 who seroconverted, in 22 (91%), ALT normalization occurred in the first year. HBV DNA levels became undetectable within 3 months after initiating Lamivudine treatment in 19 out of 21 patients (90.4%) in whom it was done. The 2 patients in whom it was still detectable, one of whom had chronic renal failure and was on maintenance peritoneal dialysis, did not attain seroconversion on long-term treatment.

### Follow up and relapse

Median follow-up of 8 months [Range 3–18 months] was done in 20 patients who had seroconverted and stopped Lamivudine. Two patients did not come for follow up after serconversion and in the other two, minimum follow up of 3 months was not available at time of analysis. Relapse was seen in 7 patients (35%), which occurred at a median of 6 months after stopping treatment [Range 3–8 months].

### Breakthrough

Breakthrough infection [i.e. re- emergence of DNA positivity, increase in viral load and increase in ALT] during treatment occurred in 6 patients out of the 25 (24%) who did not achieve HBeAg loss. These 6 had undetectable DNA levels initially at 3 months after starting Lamivudine. The onset of viral breakthrough was at 10 months in 2, 15 months in 1, 18 months in 1, and at 27 months in 2. None of these patients achieved seroconversion on long-term Lamivudine and were persistently HBeAg positive.

## Discussion

The present study in Indian patients show high seroconversion rates in the first year (28.6%), reaching 40% at end of 3 years. The first year rate is higher than previous Western and Asian studies but by the 3^rd ^year the cumulative rate becomes similar to that mentioned by Leung NW et al [16]. The rate of HBeAg loss, cumulatively rising from 36.6% in the first year to 58.3% by third year is also more than that showed by Perrillo RP et al, 25% in the first year [13] and Dienstag JL et al, 32%[12]. These results clearly indicate that Indian patients have higher HBeAg loss and seroconversion rates in the first year of treatment but the cumulative response seen on extended treatment is not much and by 3 years, the seroconversion rate reaches only 40%, which is mentioned in the previous studies. Seroconversion rates were further enhanced in patients with elevated pretreatment ALT, reaching 60% by 3 years in those with baseline ALT>2 × ULN. The 1^st ^year seroconversion rate in these patients is higher than that seen by Leung NW et al (27%) but again; by the 2^nd ^and 3^rd ^years the rates become similar [16]. It is also seen that the maximum percentage of seroconversions occurred in the first year (70.8% of all seroconversions), which is more than that seen by Leung et al, 56.5% [16]. It is not clear why the seroconversion rate in Indian patients, as seen in the present study is different from the previous published Western and Asian rates. The baseline median ALT concentration in the present study (1.8 × ULN) is comparable to that in previous studies, [14,16] and less than reported by Perrillo et al (2.2 × ULN)[13]. Also taking only those with elevated ALT, the 1^st ^year seroconversion rate in the in the present study is still higher than that mentioned in previous studies [12,16]. The mean HBV DNA level (920 mEq/ml) is actually more than that seen by Lau et al (587 mEq/ml)[15]. Therefore differences in these baseline characteristics that influence seroconversion are not the reason for the higher seroconversion rate seen. Differences in the HBV genotype can account for changing patterns of HBV response to treatment, in different geographic locations and is mentioned later.

The effect that progressively higher levels of pretreatment ALT had on HBeAg loss was striking. Previous studies with Lamivudine have shown that there is a significant correlation between pretherapy ALT levels and HBeAg seroconversion as well as HBeAg loss. Among patients with pretreatment ALT 2–5 × ULN, 70% achieved HBeAg loss and at the highest level (> 5 × ULN), 80% experienced HBeAg loss. This is much more than seen by Chien RN et al -64%[14] and Perrillo RP et al; 56%[13], but similar to that seen by Liaw YF et al; 80%[23]. As the number of patients with ALT levels greater than 5 times the ULN was relatively small, a larger sample size would be required for a more accurate estimate. As ALT elevations in patients with chronic hepatitis B are the results of T-cell -mediated hepatocytolysis, [24] the level of ALT elevation reflects the level of T-cell immune response of the patients to HBV. As also shown by the present analysis, antiviral agents like Lamivudine are more effective, in terms of HBeAg seroconversion, in patients who have mounted an ongoing immune response to HBV. The high HBeAg seroconversion in patients with high baseline ALT levels seems to the result of a concerted effort of (1) the immune -mediated killing of the hepatocytes harboring cccDNA by the antiviral defenses of the host, and (2) the potent direct antiviral effect of Lamivudine and the enhanced CD-4 responses resulting from Lamivudine therapy. This suggests that alternative treatment strategies need to be defined for patients in the immune tolerance phase or with a low anti-HBV immune response. The present study also showed significant correlation between low baseline HBV-DNA levels and HBeAg loss. This has been shown in previous studies [11-15]. That the other baseline factors like age, sex, BMI and weight had no effect on seroconversion has also been seen in previous studies [13,14].

Some studies have shown that the presence of cirrhosis could be a predictor of HBeAg sero-conversion [13,14]. As the number of patients with cirrhosis was high (38 %) in the present study as compared to previous studies, we had a doubt whether it was contributing to the high sero-conversion rates seen. But on analysis, in the present study, presence of cirrhosis had no statistical significant correlation with HBeAg loss.

Normalization of ALT was seen to occur more significantly in patients who had HBeAg loss than in those without, as seen in previous studies [15,16,23]. A disturbing finding in the present study was the high relapse rate of 35% post treatment. This is in variance with Western studies showing durability of HBeAg seroconversion post treatment [12,18]. However studies from South East Asia have reported similar high relapse rates [19,20]. The cause of the high relapse rate is not clear. It may be caused by immune tolerance, which is caused by a long-standing viral infection [25,26]. It has been suggested that Lamivudine treatment can restore immune response to HBV with reduction in viral load [27]. However, long-standing infections in vertically transmitted patients may make this immune response incomplete [25,26]. Even in patients with spontaneous HBeAg seroconversion, frequent relapses were observed in patients with long-standing HBV infection [28]. The duration of additional Lamivudine treatment after HBeAg seroconversion and pretreatment HBV-DNA levels are 2 independent predictive factors for relapse [19]. Although Lamivudine can inhibit viral replication, it cannot eliminate covalently closed circular DNA (cccDNA) in hepatocytes [29]. Studies using in vitro and in vivo model systems have shown that chronic infection is maintained by the cccDNA in hepatocytes [29]. The minimum half-life of the infected cells was estimated to exceed 10 to 100 days. Therefore it was suggested that prolonged treatment over 12 months might be needed till viral clearance, otherwise the chances for relapse [30].

Although the HBeAg seroconversion rate was high in our study, it was not durable. This observation quite contrasts with results in Western countries, in which the therapeutically induced seroconversion is usually maintained [12,18]. Similar high relapse rates were seen in the study by Song et al in Korean patients [19]. The cause of the high relapse rate after HBeAg seroconversion is not clear. It may be caused by immune tolerance, which is caused by a long-standing viral infection [25,26]. It has been suggested that lamivudine therapy can restore immune response to HBV with reduction of viral load. However, long standing infections in vertically transmitted patients may make this immune response incomplete [25,26]. Even in patients with spontaneous HBeAg seroconversion, frequent relapses were observed in patients with long-standing HBV infection [28]. Therefore, it can be suggested that HBeAg seroconversion does not necessarily guarantee prolonged suppression of HBV infection in those endemic areas in which perinatal transmissions are common.

Viral breakthrough was seen in 6 patients. In all of these six, HBV DNA had become undetectable initially at 3 months. All six did not achieve seroconversion with Lamivudine treatment. Sequencing of HBV genome for YMDD mutations was not performed to confirm viral resistance emerging. Resistance to Lamivudine typically develops after 6 months of treatment and is associated with mutations in the highly conserved catalytic region of the HBV polymerase gene [15]. Previous studies show the development of resistance in a high proportion of patients [31,32]. In studies from Asia, resistance was reported to occur in 17% of patients after 1 year [11], 26% after 2 years [33] and 49% after 3 years of treatment [34]. In the Indian study by Wakil et al, frequency of emergence of YM5521/VDD mutations was 29% and presence of normal ALT and low levels of HBV-DNA did not exclude the existence of resistant mutants [35]. Therefore only looking at biochemical and virologic breakthrough, as in the present study will miss out on identifying the emergence of viral resistance in most. The majority of patients in these studies, who developed resistance, still had biochemical and virologic evidence of improvement in the liver disease [23,33,34].

The genetic heterogeneity of the HBV genome has been established and seven genotypes (A to G) can be classified, based on comparison of complete HBV genomes [36]. The geographic distribution of these genotypes is heterogeneous with genotypes A and D being more common in India [39]. Studies are now coming out showing differences in the natural history and response to treatment among the various genotypes. Studies have shown that genotype C is associated with more severe liver injury as compared with genotype B [37]. In a study by Yuen MF et al it was seen that genotype C was associated with lower rate of HBeAg seroconversion whereas genotype B had earlier onset of seroconversion [21]. HBV genotype has also been related to interferon treatment. In a study on German subjects, interferon induced HBeAg seroconversion was higher among patients with genotype A than those with genotype D [40]. Another report from Taiwan found that the rate of HBeAg loss was significantly higher in patients with genotype B compared with C [38]. A third study in HBeAg negative patients found that patients with genotype A responded better than genotype D (70% vs. 40%) [41]. With regards to Lamivudine, reports are less and only one study mentions better response of genotype B as compared to genotype C [42]. Therefore geographic differences in the natural history and response to treatment of chronic hepatitis B could be explained by the genotypic variations of HBV between different geographic regions. This may explain the higher early sero-conversion rates seen in this present study as compared to other Western and Asian studies. Also whether any host genetic factor could be influencing the rate of seroconversion has to be ascertained. Future work has to address these issues.

## Conclusion

The results show that the HBeAg seroconversion rate in the first year in Indian patients is higher than that published from previous Western and Asian studies, but by three years the seroconversion rates become similar. Maximum seroconversion occurs in the first year with not much additional benefit on continuing treatment into the 2^nd ^and 3^rd ^years. Pretreatment ALT and HBV DNA levels were significant predictors of HBeAg loss. Relapse rates after treatment were high and were comparable to previous Asian studies. Future work has to done to elucidate the cause for the geographic variations in the rates of HBeAg seroconversion, especially with regard to influence of genotypes and any host genetic factors identified.

## Abbreviations

CHB: chronic hepatitis B

HBeAg: hepatitis B e antigen

ALT: alanine amino-transferase

HBV-DNA: hepatitis B virus DNA

## Competing interests

The author(s) declare that they have no competing interests.

## Authors' contributions

GA: Participated in the study design, collection of data and drafting the manuscript

CSB, KS, TSN: Participated in the study design and collection of data

GC: Conceived the study, participated in its design, coordination, drafting the manuscript

## Pre-publication history

The pre-publication history for this paper can be accessed here:



## Figures and Tables

**Table 1:** Base-line Characteristics of the Patients

Age (yr)	
Median	40
Range	4–80
Male sex (%)	
Weight (kg)	83.3
Median	58.5
Range	14–101
Route of HBV acquisition (%)	
Blood Transfusion	11.7
Renal Dialysis	13.3
Sexual	5
Perinatal	3.3
Others	5
Unknown	61.7
BMI (kg/m2)	
Median	22.8
Range	16.4–29.5
ALT (U/L)	
Abnormal (%)	83.3
Median	72
Range	27–394
Median × ULN	1.8
HBV-DNA (mEq/ml)	
Mean	920
Range	0.8–4500
Cirrhosis (%)	38.33

**Table 2:** Pre-treatment Variables Influencing HBeAg Loss

Variable	HBeAg Loss (n = 35)	No HBeAg Loss (n = 25)	p value
Age (years)	37.5	44	ns
			
Sex			
Male	29	21	ns
Female	6	4	
			
Weight (Kg)	60	55	ns
			
BMI	23.1	22.7	ns
			
Median ALT (U/L)	94	45	0.002
Range	32–394	27–254	
			
Median HBV-DNA (mEq/ml)	111.3	958	0.004
Range	0.8–4500	4.2–3500	
			
Cirrhosis			
Present	13	10	ns
Absent	22	15	
			
ALT Normalization			
Yes	23	5	0.02(df = 1)
No	9	13	

**Table 3:** HBeAg Seroconversion and Loss by Pretreatment ALT Level

	HbeAg Seroconversion		HBeAg Loss	
ALT Level	Number	%	Number	%

<= 1 × ULN	0/10	-	3/10	30
>1-<=2 × ULN	6/20	30	10/20	50
>2-<=5 × ULN	11/20	55	14/20	70
> 5 × ULN	7/10	70	8/10	80
Total	24/60	40	35/60	58.3
